# Influence of the type of volatile anesthetic on the development of acute kidney injury after endovascular repair of aortic aneurysm: a randomized controlled trial (DESEVAR study)

**DOI:** 10.3389/fphar.2025.1660359

**Published:** 2025-09-25

**Authors:** A. Arnalich-Montiel, I. M. Barrio-Pérez, A. Burgos-Santamaría, C. Fernández-Riveira, A. Lázaro, M. A. González-Nicolás, J. Río, J. M. Bellón, M. I. Canal, J. M. Ligero, B. Quintana-Villamandos

**Affiliations:** ^1^ Department of Anesthesia and Intensive Care, Hospital General Universitario Gregorio Marañón, Madrid, Spain; ^2^ Department of Pharmacology and Toxicology, Faculty of Medicine, Universidad Complutense de Madrid, Madrid, Spain; ^3^ Department of Nephrology, Renal Physiopatology Laboratory, Instituto de Investigación Sanitaria Gregorio Marañón, Hospital General Universitario Gregorio Marañón, Madrid, Spain; ^4^ Department of Physiology, School of Medicine, Universidad Complutense de Madrid, Madrid, Spain; ^5^ Department of Angiology and Vascular Surgery, Hospital General Universitario Gregorio Marañón, Madrid, Spain; ^6^ Department Statistics, Instituto de Investigación Sanitaria Gregorio Marañón, Madrid, Spain

**Keywords:** aortic aneurysm, desflurane, sevoflurane, EVAR, acute kidney injury, NGAL

## Abstract

Acute kidney injury (AKI) frequently occurs after endovascular aortic aneurysm repair (EVAR), leading to significant morbidity and mortality. It is associated with contrast administration, hypovolemia, arterial microembolization due to renal artery manipulation, ischemia–reperfusion syndrome, and other patient comorbidities. However, little is known about the effect of volatile anesthetics on the development of AKI in this context. Therefore, we aimed to investigate renal function in patients anesthetized with desflurane or sevoflurane for EVAR. For this, we conducted a single-center randomized clinical trial involving 80 patients scheduled for elective EVAR under general anesthesia. Patients were randomly assigned to the desflurane or sevoflurane anesthesia group. Biochemical variables of kidney function and biomarkers for AKI were studied at three different time points: before surgery, after surgery, and 24 h after surgery. Plasma creatinine, cystatin C, estimated glomerular filtration, uric acid, and kidney injury molecule-1 did not significantly change between both groups. A mixed linear model demonstrated a significant interaction (p = 0.01) of plasma neutrophil gelatinase-associated lipocalin (NGAL) between sevoflurane and desflurane. Both groups showed a progressive increase in plasma NGAL (sevoflurane 3.713 ng/mL, p < 0.001 and desflurane 1.774 ng/mL, p < 0.001) when comparing the moment before surgery with respect to 24 h after surgery. However, sevoflurane caused a higher plasma NGAL concentration than desflurane after 24 h of surgery (8.66 ± 5.09 ng/mL vs. 6.51 ± 3.86 ng/mL, P = 0.03). Desflurane was associated with a lower postoperative AKI than sevoflurane in patients undergoing EVAR. Further research is required to corroborate our results and evaluate the clinical importance. Trial registration: EudraCT: 2016-003906-16; ClinicalTrials.gov: NCT03917186.

## 1 Introduction

Acute kidney injury (AKI) remains a major problem for patients undergoing endovascular aortic aneurysm repair (EVAR). It is a postoperative complication with a multifactorial etiology, mainly associated with intraoperative contrast administration, hypovolemia, renal microembolization, complications directly related to renal artery manipulation, lower limb ischemia (ischemia reperfusion syndrome), and multiple other comorbidities typically observed in patients undergoing vascular surgery. All of this have a negative impact on the outcomes of vascular patients, leading to a significant increase in their mortality (De Paulis et al., 2022; Zambetti et al., 2024).

Volatile anesthetics (desflurane and sevoflurane) are commonly used in clinical practice (during induction and maintenance of general anesthesia) (Wang et al., 2021). Previous studies have shown that both desflurane and sevoflurane present organ protection effects (Wu et al., 2014). However, no beneficial effects on perioperative renal function have been reported so far in patients undergoing EVAR.

Volatile anesthetics produce changes in renal function due to their action on the cardiovascular system and the autonomic nervous system (Iguchi et al., 2019; Osborn and Cruz-Lynch, 2021). Some of them can also produce direct renal toxicity due to metabolism (production of inorganic fluoride ions) and degradation processes (production of toxic products, such as compound A, when interacting with carbon dioxide absorbents within the anesthesia circuit) (Kharasch, 2008). Preclinical studies (in rats) (Gonsowski et al., 1994; Obata et al., 2000) have shown nephrotoxicity (renal proximal tubular injury) after administering sevoflurane (under low fresh gas flow conditions), which is directly related to production of compound A. However, it has not been demonstrated that compound A affects human renal function (Conzen et al., 1995; Sondekoppam et al., 2020; Park et al., 2022). A meta-analysis of randomized trials revealed the safety of sevoflurane regarding renal function (Ong Sio et al., 2017). Unlike sevoflurane, degradation of desflurane does not produce component A (Ebert and Arain, 2000).

The aim of this clinical trial was to examine the effects of desflurane or sevoflurane anesthesia on renal function when administered during EVAR, using clinical and biochemical parameters (markers of kidney injury in plasma), before surgery, after surgery, and 24 h postoperatively. We hypothesized that desflurane could be superior to sevoflurane with respect to renal function in patients with aortic aneurysm undergoing endovascular repair.

## 2 Methods

### 2.1 Study design and approval

This study was conducted at Gregorio Marañón General University Hospital (between 2017 and 2021) as a randomized phase IV clinical trial, with two parallel groups (desflurane and sevoflurane). It was performed in accordance with the principles of the Declaration of Helsinki and approved by the Ethics Committee of our institution, the Spanish Medical Products Agency (EudraCT 2016-003906-16), and was registered at *Clinicaltrials. gov* (NCT03917186).

### 2.2 Patient enrollment, randomization, and blinding

Patients (male or female) aged ≥18 years old with aortic aneurysm and undergoing elective EVAR were included in the study (only standard bifurcated stent-grafts without branches or fenestrations were included). Prior to the inclusion, informed consent was obtained from all the subjects. Exclusion criteria were patient refusal to participate in the study or clinical history with any contraindication for volatile anesthetic administration (desflurane or sevoflurane).

A total of 80 patients were consecutively recruited and randomized in a 1:1 allocation (using EPIDAT 3.1 software, the codes were kept in sealed envelopes, and these envelopes were provided to the anesthetist responsible for intraoperative care) to receive either sevoflurane (n = 40) or desflurane (n = 40) during the maintenance of general anesthesia. Patients and investigators (including staff responsible for the analysis of biological samples) were blinded to the volatile anesthetics used (desflurane or sevoflurane). However, it was not possible to blind the anesthesiologist responsible for the intraoperative management of the patient, due to the differences between the vaporizer used for each volatile anesthetic.

### 2.3 Study protocol

All patients followed the same anesthetic protocol. Patients were monitored using electrocardiograms, invasive arterial pressure, and cardiac index (Sistema FloTrac/Vigileo^®^, Edwards Lifesciences S.L., Spain) via radial artery catheterization, pulse oximetry, capnography, peripheral quantitative neuromuscular status, bispectral index (Monitor BIS^®^, Aspect Medical Systems™ Inc., Natick, MA), and hourly diuresis (with a urinary catheter inserted in the urethra). General anesthesia was induced with i. v. propofol (2–3 mg/kg), fentanyl (2 μg/kg), and rocuronium (0.6–1 mg/kg).

For the maintenance phase of anesthesia, desflurane or sevoflurane was administrated and titrated (following the instructions of the technical datasheet) to maintain an adequate hypnotic depth (bispectral index target range between 40 and 60), in combination with continuous i.v. infusion of remifentanil (0.1–0.5 μg/kg/min), and additional rocuronium boluses (0.25 mg/kg) were administered as appropriate for the surgeons’ requirements. Parameters applied during ventilation were as follows: volume-controlled ventilation, tidal volume of 8 mL/kg (ideal weight), FiO2 ≥ 0.4, respiratory rate to maintain an end-tidal carbon dioxide between 35 and 45 mmHg, and fresh gas flow above 2 L/min. Hemodynamic management was performed according to the protocol previously described (Pestaña et al., 2014) (to maintain a mean arterial pressure ≥65 mmHg and a cardiac index ≥2.5 L/min/m^2^), and for intravascular volume maintenance, crystalloids were infused at 2–4 mL/kg/h.

After the surgery, acetaminophen was administered, and if necessary, the neuromuscular blockade was reversed with sugammadex. After extubation in the operating room, all patients were transferred to the postoperative care unit.

### 2.4 Sample and measurement methods

Arterial blood samples were collected in all patients at three different study time points: before surgery (before the anesthesia induction), immediately after surgery, and 24 h postoperatively.

Biochemical variables of kidney function were collected and analyzed by Clinical Chemistry from Gregorio Marañón University Hospital Research Institute.

Urine samples were collected under sterile conditions and refrigerated until processing. They were centrifuged at 2,500 rpm for 15 min, aliquoted into cryovials suitable for freezing, and stored at −80 °C.

EDTA tube blood samples were placed on ice and then centrifuged (4 °C, 2000 g for 15 min). Plasma was stored in a −80 °C freezer until renal biomarkers of AKI were analyzed. In particular, we studied the following: *neutrophil gelatinase-associated lipocalin* (NGAL) *and kidney injury molecule-1* (KIM-1). The Laboratory of Renal Pathophysiology (from the Unit of Experimental Medicine and Surgery of Gregorio Marañon University Hospital, IiSGM) analyzed KIM-1 and NGAL using commercial immunoassay kits: *Human KIM-1* (Kidney Injury Molecule 1) *ELISA Kit* (Elabscience Biotechnology Inc., Houston, TX, United States) and *Human NGAL ELISA Kit* (Elabscience Biotechnology Inc., Houston, TX, United States), in accordance with the manufacturers’ instructions.

### 2.5 Statistical methods

Categorical variables were described as frequencies (percentages) and were compared using chi-square test or Fisher exact test as required. Continuous variables were presented as the mean ± standard deviation (SD). Normality was assessed using the Kolmogorov–Smirnov test. To study the differences between the means of each group, parametric tests (independent Student’s t-test and paired t-test) were used (for normally distributed data and appropriate number of patients in each group). The evolution and differences between the two study groups (desflurane and sevoflurane) at the three different time points of the study were estimated using linear mixed models, considering the individual as a random effect and time, group, and time–group interaction as fixed effects. A P-value of less than 0.05 was considered statistically significant. Statistical analyses were performed using *SPSS software 25.0* (Armonk, NY: IBM Corp.) and *Stata Software 18* (College Sation, StataCorp. LLC).

## 3 Results

A total of 80 patients undergoing EVAR completed the study: 40 patients in the desflurane group and 39 in the sevoflurane group suffered from abdominal aortic aneurysm, whereas one patient with thoracic aortic aneurysm was enrolled in the sevoflurane group.

### 3.1 Preoperative patient characteristics and intraoperative data

Clinical characteristics of the patients are shown in Table 1. No significant differences were observed between groups regarding demographic data (gender, age, weight, and height), anesthetic risk score of the American Society of Anesthesia (ASA), cardiovascular risk factors (smoking, diabetes mellitus, hypertension, and dyslipidemia), end-organ damage/failure (heart disease, stroke, and chronic kidney disease), and chronic treatment (calcium channel blockers, angiotensin-converting enzyme inhibitors, angiotensin receptor blockers, β-blockers, diuretics, statins, oral hypoglycemic agents, and antiplatelet therapy).

**TABLE 1:** Baseline characteristics in the desflurane and sevoflurane groups.

	Desflurane (n = 40)	Sevoflurane (n = 40)	p-value
Age (yr)	75.75 ± 6.99	76.35 ± 5.62	0.67
Sex (male)	40 (100%)	39 (97.5%)	1.00
Height (cm)	169.63 ± 7.58	170.75 ± 6.99	0.49
Weight (kg)	85.05 ± 15.44	81.34 ± 12.42	0.24
ASA physical status			0.513
II	2 (5%)	3 (7.5%)	
III	38 (95%)	35 (87.5%)	
IV	0 (0%)	2 (5%)	
Hypertension	33 (82.5%)	35 (87.5%)	0.53
Diabetes	13 (32.5%)	8 (20%)	0.20
Smokers	9 (22.5%)	10 (25%)	0.79
Dyslipidemia	27 (67.5%)	23 (57.5%)	0.35
Heart disease	20 (50%)	19 (47.5%)	0.82
Cerebrovascular accident	5 (12.5%)	3 (7.5%)	0.71
Chronic kidney disease	6 (15%)	7 (17.5%)	0.76
Treatment			
CCB	10 (25%)	8 (20%)	0.59
ACEi	17 (42.5%)	13 (32.5%)	0.35
ARB	9 (22.5%)	7 (17.5%)	0.57
β-blocker	12 (30%)	13 (32.5%)	0.80
Diuretics	14 (35%)	9 (22.5%)	0.21
Statin	32 (80%)	28 (70%)	0.30
OHA	11 (27.5%)	7 (17.5%)	0.28
Antiplatelet	23 (57.5%)	22 (55%)	0.82

ACEi, angiotensin-converting enzyme inhibitor; ARB, angiotensin receptor blocker; ASA, American anesthesiologists physical status scale; CCB, calcium channel blocker; OHA, oral hypoglycemic agent. Values are expressed as mean ± SD or frequencies (%).

Intraoperative variables in the desflurane and sevoflurane groups are summarized in Table 2. Both groups were similar in terms of the duration of anesthesia (defined as the time from patient monitoring to their extubation in the operating room), surgical time (defined as the time from skin incision to the closure of the surgical wounds), and duration of volatile anesthetic administration (desflurane or sevoflurane). Intraoperatively, all patients received the same type of the intravenous contrast agent (iopamidol), and there were no significant differences between groups in the volume of the drug administrated. Intraoperative data included administered fluids, the use of vasoactive drugs (phenylephrine and ephedrine), and quantification of transfusion requirements (red blood cell concentrates), not finding significant differences between both groups, i.e., desflurane and sevoflurane.

**TABLE 2:** Intraoperative anesthesia and surgical data in the desflurane and sevoflurane groups.

	Desflurane (n = 40)	Sevoflurane (n = 40)	p-value
Surgical time (min)	182.38 ± 79.16	156.55 ± 59.31	0.10
Anesthesia time (min)	250.28 ± 87.15	220.90 ± 64.83	0.09
Inhaled anesthetic time (min)	230.93 ± 86.80	197.85 ± 61.91	0.053
Contrast volume (mL)	166.10 ± 105.43	137.21 ± 61.48	0.14
Total fluids (mL)	1,350.35 ± 627.89	1,129.38 ± 484.33	0.08
Phenylephrine/ephedrine	37 (92.5%)	33 (82.5%)	0.17
Transfusion (RBC)	1 (2.5%)	3 (7.5%)	0.61

RBC, red blood cell concentrates. Values are expressed as mean ± SD or frequencies (%).

Once surgery was finished, all patients were extubated in the operating room. It was found that the duration of stay in the postoperative care unit (min) was not influenced by the choice of the anesthetic agent, desflurane or sevoflurane (1,110.68 ± 262.26 and 1,198.60 ± 454.09 respectively, p = 0.29).

### 3.2 Desflurane and sevoflurane on renal function

**FIGURE 1:**
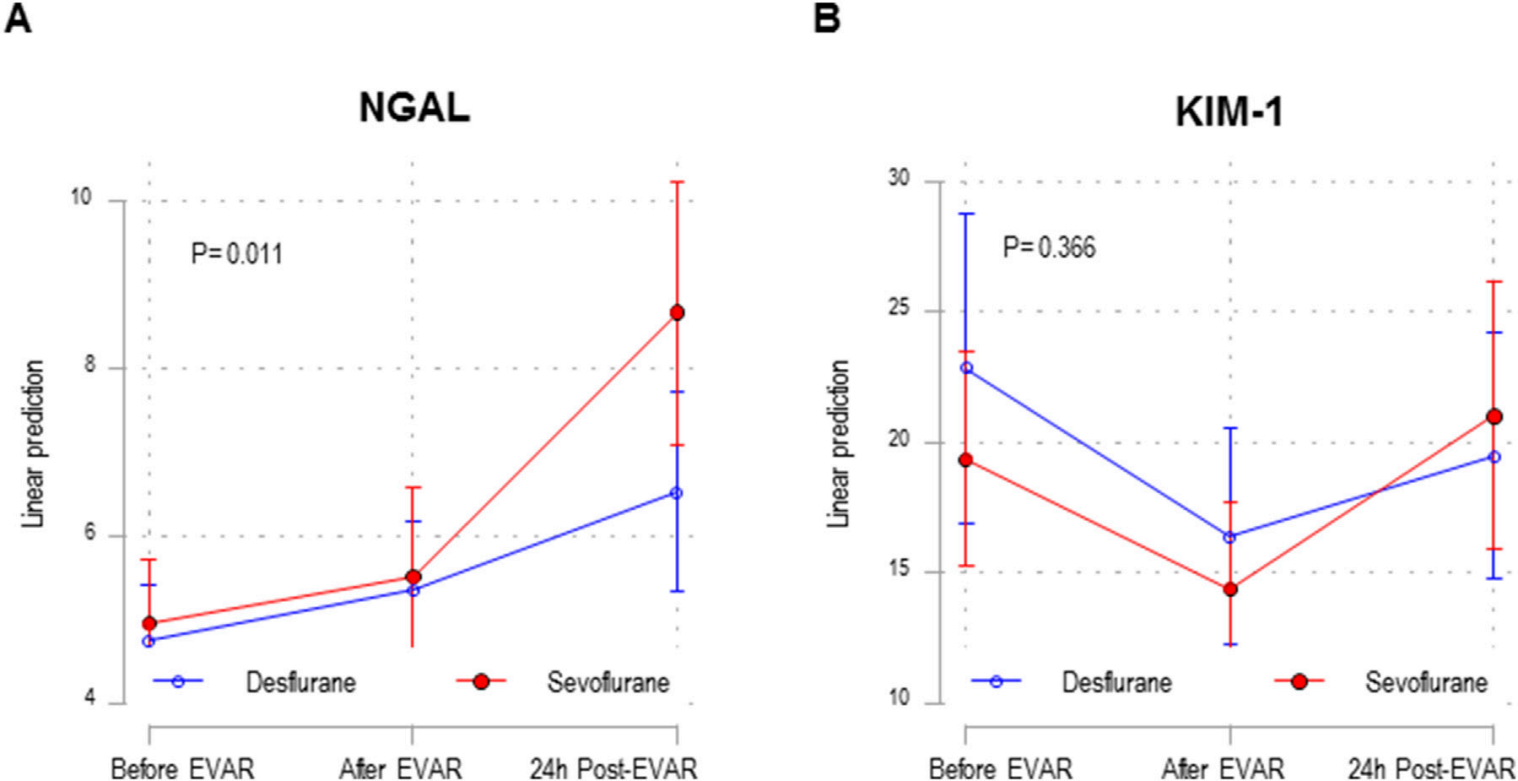
Mixed linear model in NGAL **(A)** and KIM-1 **(B)** study in the desflurane (n = 40) and sevoflurane (n = 40) groups, at the three study time points. EVAR, endovascular aneurysm repair. Estimated NGAL means and their 95% confidence intervals obtained using the linear mixed model. The p-value in the figure corresponds to the interaction between the anesthetic group and measurement time points.

Renal outcomes were compared between desflurane and sevoflurane groups (Table 3; Figures 1A,B). There were no significant differences in renal function markers (plasma creatinine, cystatin, and uric acid concentrations) or diuresis between the two groups at any of the three time points of the study. In addition, when the estimated glomerular filtration rate (eGFR) calculated by *Chronic Kidney Disease Epidemiology Collaboration* (CKD-EPI) was assessed, no differences were observed between patients receiving desflurane or those receiving sevoflurane.

**TABLE 3:** Indicators’ renal function status in the desflurane and sevoflurane groups, at three time points of the study.

	Desflurane (n = 40)	Sevoflurane (n = 40)	p-value
Plasma cystatin C (mg/L)			
Before EVAR	1.21 ± 0.34	1.22 ± 0.29	0.868
After EVAR	1.21 ± 0.40	1.16 ± 0.30	0.589
24 h postoperatively	1.22 ± 0.46	1.23 ± 0.36	0.919
eGFR cystatin (mL/min/1.73 m2)			
Before EVAR	61.7 ± 17.61	59.92 ± 17.14	0.649
After EVAR	63.55 ± 21.42	64.40 ± 19.31	0.853
24 h postoperatively	63.87 ± 21.44	60.44 ± 19.74	0.467
Plasma creatinine (mg/dL)			
Before EVAR	0.93 ± 0.30	0.95 ± 0.21	0.691
After EVAR	1.01 ± 0.34	0.94 ± 0.24	0.304
24 h postoperatively	1.01 ± 0.41	0.95 ± 0.27	0.445
eGFR creatinine (mL/min/1.73 m2)			
Before EVAR	77.97 ± 16.49	75.33 ± 15.46	0.464
After EVAR	73.54 ± 16.75	76.09 ± 16.19	0.490
24 h postoperatively	75.31 ± 18.50	76.18 ± 19.06	0.837
Uric acid (mg/dL)			
Before EVAR	6.02 ± 1.41	5.89 ± 1.38	0.684
After EVAR	5.70 ± 1.32	5.67 ± 1.28	0.918
24 h postoperatively	5.77 ± 1.38	5.67 ± 1.25	0.742
Diuresis (mL)			
Before EVAR	—	—	—
After EVAR	361.23 ± 338.06	346.38 ± 276.78	0.858
24 h postoperatively	1,048.65 ± 510.19	1,267.95 ± 530.79	0.071
Plasma KIM-1 (ng/mL)			
Before EVAR	22.86 ± 19.31	19.34 ± 13.39	0.341
After EVAR	16.39 ± 13.46	14.38 ± 10.75	0.457
24 h postoperatively	19.48 ± 15.36	21.02 ± 16.64	0.665
Plasma NGAL (ng/mL)			
Before EVAR	4.74 ± 2.18	4.95 ± 2.44	0.685
After EVAR	5.34 ± 2.64	5.50 ± 3.45	0.815
24 h postoperatively	6.51 ± 3.86	8.66 ± 5.09	0.032

eGFR cystatin C, estimated glomerular filtration rate based on cystatin C; eGFR creatinine, estimated glomerular filtration rate based on creatinine; EVAR, endovascular aneurysm repair; KIM-1, kidney injury molecule 1; NGAL, neutrophil–gelatin-associated lipocalin. Values are expressed as mean ± SD.

Furthermore, we conducted linear mixed modeling for biomarkers of AKI (KIM-1 and NGAL). Analyzing the KIM-1 biomarker, the linear mixed model showed a non-significant interaction (p = 0.36) between the two study groups, desflurane vs. sevoflurane (after surgery vs. before surgery, p = 0.63; 24 h postoperatively vs. before surgery, p = 0.17) (Figure 1B). KIM-1 did not show significant differences when comparing both groups, desflurane and sevoflurane, at the three study time points (Table 3). However, the linear mixed model for NGAL shows a significant interaction (p = 0.01) between the two study groups, desflurane vs. sevoflurane (after surgery vs. before surgery, p = 0.90; 24 h postoperatively vs. before surgery, p = 0.005) (Figure 1A). Both groups showed a progressive increase in plasma NGAL before surgery (sevoflurane 3.713 ng/mL, p < 0.001 and desflurane 1.774 ng/mL, p < 0.001) comparing to 24 h after surgery. However, after 24 h of surgery, the increase in NGAL was significantly higher in the sevoflurane group than in the desflurane group (8.66 ± 5.09 vs. 6.51 ± 3.86 ng/mL, p = 0.03) (Table 3).

## 4 Discussion

The main result of the present study is that desflurane was associated with a lower plasma NGAL concentration (biomarker of AKI) in patients undergoing EVAR, compared to sevoflurane, as we had previously hypothesized. Both groups (desflurane and sevoflurane) showed a progressive increase in NGAL when comparing the moment before surgery with respect to 24 h postoperatively; however, the sevoflurane group had a higher plasma NGAL concentration than the desflurane group.

Postoperatively, AKI is related with an increased risk of morbidity and mortality (Coca et al., 2012). A recent meta-analysis revealed a lower incidence of AKI with intravenous anesthesia than with volatile anesthesia (Franzén et al., 2023). Based on changes in creatinine and cystatin C, sevoflurane is associated with an increased risk of renal dysfunction after cardiovascular surgery, compared with propofol (Yoo et al., 2014). However, there were no significant differences in plasma creatinine and NGAL measurements between desflurane and propofol (only a significant increase in NGAL was observed between baseline and 2 h postoperatively in the propofol group) (Guerrero-Orriach et al., 2022; Yoon et al., 2025). Therefore, some volatile anesthetic drugs might be more suitable for the kidney than others.

Nowadays, desflurane and sevoflurane are the volatile anesthetics of choice for general anesthesia (Wang et al., 2021). A limited number of studies have investigated the effects of desflurane and sevoflurane on kidney function in patients undergoing surgery, not finding significant differences between the effects of both agents (Kim et al., 2013; Abou Hussein et al., 2015; Karadeniz et al., 2017; Duymaz et al., 2017; Lee et al., 2019; Ebert and Arain, 2000; Ayanoğlu Taş et al., 2022). Among the considerations that could potentially explain these mixed results regarding postoperative kidney function are the small sample size of the studies, the use of standard biomarkers with low sensitivity to detect AKI, and the limited reports on their clinical outcomes.

To our knowledge, this is the first study that has shown the potential association between the intraoperative use of desflurane and a lower increase on a sensitive biomarker of AKI such as NGAL in patients undergoing EVAR, compared to that observed in patients receiving sevoflurane. These results are consistent with those observed in a randomized clinical trial that demonstrated a better postoperative renal function in patients who underwent hepatectomy under general anesthesia with desflurane than those with sevoflurane (Ko et al., 2010). In this study, serum creatinine was significantly higher and the eGFR was significantly lower on the third postoperative day in the sevoflurane group; however, in our clinical trial, these levels of biomarkers did not show significant differences in desflurane versus sevoflurane administration. Serum creatinine is a traditional biomarker used to estimate renal function, which is dependent of glomerular filtration, age, gender, muscle metabolism, diet, treatment, and hydration (Seijas et al., 2014). Another potential disadvantage is that it is not an early biomarker of AKI (a decrease in eGFR of 50% is necessary to produce a noticeable increase in serum creatinine) (Bagshaw and Bellomo, 2007), and in our study, creatinine was only evaluated at 24 h (although perioperative AKI can evolve over 48–72 h). These conditions could explain the different results of the study by *Ko JS et al.* with respect to the present study regarding postoperative serum creatinine (Ko et al., 2010).

Cystatin C is a biomarker of AKI, which has a higher sensitivity for detection of minor renal damage than creatinine, revealing the onset of renal injury 1 or 2 days earlier compared to creatinine (Herget-Rosenthal et al., 2004; Lisowska-Myjak, 2010). However, an increase in cystatin has also been observed in specific populations such as men, elderly patients, smokers, obese individuals, patients receiving immunosuppressive therapy, and those with thyroid gland disease (Knight et al., 2004). No significant differences were found between desflurane and sevoflurane groups in our study (neither immediately after surgery nor 24 h postoperatively) regarding serum cystatin C and eGFR. There are differences between our findings and those from other authors (Abdelhamid et al., 2013) who showed a significant increase in cystatin 24 h after EVAR surgery; however, this could potentially be explained because the mean of cystatin C was higher in these patients before surgery than the normal range, having not appreciated this difference in our study.

NGAL is a biochemical marker with a high sensitivity and specificity for AKI detection from the very early stages (it increases 2 h after renal injury and 24 h earlier compared to the increase in creatinine). The increase in NGAL indicates tubular damage (Mishra et al., 2005; Wagener et al., 2006; Bennett et al., 2008; Haase et al., 2009; Seijas et al., 2014) and has previously been studied in patients undergoing EVAR (Rampoldi et al., 2018; Karaolanis et al., 2019; Gombert et al., 2019; Stilo et al., 2022). In our study, both desflurane and sevoflurane groups showed a progressive and significant increase in plasma NGAL when comparing the moment before surgery with respect to 24 h after surgery. Registry analyses and clinical trials showed postoperative AKI in patients undergoing EVAR (EVAR Trial Participants, 2005; Hua et al., 2005; Lederle et al., 2009; De Bruin et al., 2013), associated with different mechanisms that included contrast administration (associated with tubular cell toxicity) (Krasinski et al., 2020; Mun et al., 2021). In the present study, there were no significant differences in the amount of contrast medium administered between the desflurane and sevoflurane groups. However, the sevoflurane group showed a higher plasma NGAL concentration than the group anesthetized with desflurane after 24 h of surgery (the linear mixed model for NGAL showed a significant interaction between the two study groups). NGAL is known mainly as a biomarker of AKI, and its levels increase in hypertension, obesity, and diabetes (Romejko et al., 2023). This could influence the outcomes of the present study; however, we did not observe significant differences between groups regarding cardiovascular risk factors (diabetes mellitus, hypertension, and obesity). Although statistically significant (p = 0.03), we do not know the clinical relevance of the NGAL increase (2.15 ng/mL difference between groups). Therefore, this is a potentially indicative study (results may be interpreted as indicative of early tubular damage), requiring replication with a larger sample size and longer follow-up to correlate biomarker elevation with clinical AKI.

We do not know the mechanism that produces this effect; however, compound A (a nephrotoxic metabolite resulting from the degradation of sevoflurane by carbon dioxide absorbents in the anesthesia circuit) could be related to this effect. Laboratory evidence (from animal studies) supports the nephrotoxicity of sevoflurane, but this has not been demonstrated in humans (Gonsowski et al., 1994; Obata et al., 2000). Our study revealed a significant beneficial effect of anesthesia with desflurane compared to sevoflurane regarding renal function; however, future studies are required to investigate the associated mechanisms because a meta-analysis of randomized trials (six studies in humans within clinically relevant exposures, duration of anesthesia > 3 h, and low flow sevoflurane) revealed the safety of sevoflurane regarding maintenance of renal function (Ong Sio et al., 2017).

In our study, the discordance observed between NGAL and KIM-1 demonstrates that NGAL may reflect early stress signals, while KIM-1 increases in response to sustained tubular epithelial damage. In the clinical context, where different factors might affect renal perfusion (hemodynamic fluctuations, volatile anesthetics, and contrast administration), NGAL may have captured early subclinical stress, while KIM-1 might have required more established injury to increase (Yousef Almulhim, 2025).

This study has several limitations. First, in the present clinical trial, even though patients and investigators were blinded to the anesthetic used, it was not possible to blind the responsible anesthesiologist because each volatile anesthetic drug has a different vaporizer that is essential for its administration. Second, animal models have demonstrated an association between sevoflurane, component A, and renal toxicity (Gonsowski et al., 1994; Obata et al., 2000). Therefore, it would have been interesting to measure component A exposures in the present study. Third, NGAL increases rapidly within 2–6 h following renal tubular injury and remains elevated for 18–24 h in patients who develop AKI. Therefore, this early peak makes NGAL a valuable marker in the perioperative setting for detecting tubular injury before changes in serum creatinine or eGFR become evident. This early increase has a predictive value for subsequent clinical AKI in cardiac surgery and critical care patients (Côté et al., 2022; Wagener et al., 2008). This predictive capability supports the use of NGAL as a meaningful early endpoint. Nevertheless, we recognize that the lack of extended follow-up limits our ability to correlate biomarker elevation with clinical AKI. Future investigations with longer follow-up and clinical outcome assessment are needed to confirm the implications of the biomarker changes observed. Finally, this clinical trial was performed to examine the effect of desflurane or sevoflurane anesthesia on renal function when administered in EVAR after 24 h of surgery. Because AKI after EVAR causes increased morbidity and mortality (Zambetti et al., 2024), it would be interesting to follow-up patients over the long term. Future studies will be necessary to respond to this objective.

## 5 Conclusion

In our study, desflurane was associated with a lower postoperative AKI than sevoflurane in patients undergoing EVAR. Further research is required to corroborate our results and evaluate their clinical importance.

## Data Availability

The original contributions presented in the study are included in the article/supplementary material; further inquiries can be directed to the corresponding author.
